# Inhibition of Fumonisin B_1_ Cytotoxicity by Nanosilicate Platelets during Mouse Embryo Development

**DOI:** 10.1371/journal.pone.0112290

**Published:** 2014-11-10

**Authors:** Yu-Jing Liao, Jenn-Rong Yang, Shuen-Ei Chen, Sing-Jhou Wu, San-Yuan Huang, Jiang-Jen Lin, Lih-Ren Chen, Pin-Chi Tang

**Affiliations:** 1 Division of Physiology, Livestock Research Institute, Council of Agriculture Executive Yuan, Tainan, Taiwan; 2 Department of Animal Science, National Chung Hsing University, Taichung, Taiwan; 3 Agricultural Biotechnology Center, National Chung Hsing University, Taichung, Taiwan; 4 Center for the Integrative and Evolutionary Galliformes Genomics, National Chung Hsing University, Taichung, Taiwan; 5 Center of Nanoscience and Nanotechnology, National Chung Hsing University, Taichung, Taiwan; 6 Institute of Polymer Science and Engineering, National Taiwan University, Taipei, Taiwan; Soonchunhyang University, Republic of Korea

## Abstract

Nanosilicate platelets (NSP), the form of natural silicate clay that was exfoliated from montmorillonite (MMT), is widely used as a feed additive for its high non-specific binding capacity with mycotoxins such as fumonisin B_1_ (FB_1_), and has been evaluated its safety for biomedical use including cytotoxicity, genotoxicity, and lethal dosage (LD). In the study, we further examined its toxicity on the development of CD1 mouse embryos and its capacity to prevent teratogenesis-induced by FB_1_. *In vitro* cultures, NSP did not disturb the development and the quality of intact pre-implantation mouse embryos. Further, newborn mice from females consumed with NSP showed no abnormalities. NSP had an unexpected high adsorption capacity *in vitro*. In contrast to female mice consumed with FB_1_ only, a very low residual level of FB_1_ in the circulation, reduced incidence of neutral tube defects and significantly increased fetal weight were observed in the females consumed with FB_1_ and NSP, suggesting a high alleviation effect of NSP on FB_1_ *in vivo*. Furthermore, FB_1_ treatment disturbed the gene expression of sphingolipid metabolism enzymes (*longevity assurance homolog 5*, *LASS 5*; *sphingosine kinase 1*, *Sphk1*; *sphingosine kinase 2*, *Sphk2*; *sphingosine 1- phosphate lyase*, *Sgpl1*; *sphingosine 1-phosphate phosphatase*, *Sgpp1*) in the maternal liver, uterus, fetus, and placenta, but NSP administration reversed the perturbations. Based on these findings, we conclude that NSP is a feasible and effective agent for supplementary use in reducing the toxicity of FB_1_ to animals.

## Introduction

Corn and soybean meal are the primary components of animal feed. However, animal feed can also be an excellent place for mold growth to produce mycotoxins. Out of more than 300 types of mycotoxins discovered, five are the most common in animal feed: alfatoxin, deoxynivalenol, ochratoxin, zearalenone, and fumonisins [1]. Fumonisin B_1_ (FB_1_) is a mycotoxin produced by *Fusarium verticillioides* and not discovered until 1988 [2], [3]. Equine leukoencephalomalacia [4] and porcine pulmonary edema [5] are well-known diseases caused by FB_1_. Neural tube defect (NTD) is the failure of neurulation during embryogenesis [6]. In specific strains of mice, high doses of FB_1_ also increase the incidence of NTD [7], [8], [9], [10]. Furthermore, almost all the hog, broiler, and layer feeds in the world are contaminated by FB_1_ [11]. Therefore, specific measures should be undertaken to prevent these diseases.

Supplementation of mycotoxin adsorbents is one proposed solution for the high rate of feed contamination with mycotoxins. Features of the adsorbents, *i*.*e*., total charge, charge distribution, pore size, accessible surface area, and adsorption affinity to mycotoxins, are the critical points for determining the adsorption efficiency [12]. Aluminosilicates are the largest groups of mycotoxin adsorbing agents and include bentonites, montmorillonites (MMT), zeolites, and hydrated sodium calcium aluminosilicates (HSCAS). The structures of aluminosilicates are rich in negative charges, allowing them to adsorb mycotoxins in the gastrointestinal tracts of animals [13], [14].

Among the various types of aluminosilicates, MMT have layer stack structures consisting of multiple aluminosilicate sheets with irregular polygonal shapes [15]. After exfoliation with natural MMT, nanosilicate platelets (NSP) (*ca.* 80×80×1 nm) were isolated in water to produce high surface areas (*ca*. 720 m^2^/g) and multiple ionic charges per platelets (*ca*. 20,000 ions/platelet) [16], [17]. After using these unique characteristics to bind to the surface of microorganism [18], [19], [20], it was demonstrated that the growth of various strains of bacteria was completely inhibited at 0.3% (w/v) NSP by nonspecific binding [20]. Furthermore, the surface of NSP modified by silver nanoparticles could improve the antibacterial activity but merely elicit slight immune response [21]. The low cytotoxicity and genotoxicity of NSP have been verified by several methods, including comet assay, micronucleus test, and *Salmonella* gene mutation assay. A high lethal dose (LD_50_) greater than 5,700 mg/kg body weight was also observed in rats with acute oral administration of NSP [19]. Based on the strong binding ability and the low toxicity of NSP, we expect that NSP will act as a good mycotoxin adsorption agent when used as a feed additive. However, the effect of NSP on the development of embryos has not yet been reported. In this study, we evaluate the influences of NSP on the pre-implantation development of mouse embryos and the adsorption of FB_1_ by NSP via both *in vitro* and *in vivo* assays.

## Materials and Methods

### Material preparation of nanosilicate platelets

The 10% (w/v) solution of nanosilicate platelet clay in water was acquired from the Institute of Polymer Science and Engineering, National Taiwan University. The NSP clay was dispersible in deionized or distilled water.

### Ethics statement

All animal experiments in this study and the procedures for animal handling and treatments were approved by the Institutional Animal Care and Use Committee (IACUC) at the National Chung Hsing University (no. 99–83).

### Animals

Sexually mature CD1 mice at 8 weeks of age used in this study were purchased from Bio-LASCO Taiwan Co., Ltd., and they were maintained at 25°C and 60% relative humidity environment for 1 week before treatments. All the mice were fed with the same reverse osmosis water and standard feed (MFG, Oriental Yeast Japan Co., Ltd.).

### Collection and *in vitro* culture of mouse embryos

Superovulation of female CD1 mice was induced by intraperitoneal injection of 10 IU pregnant mare serum gonadotropin (PMSG; Sergona X09; China Chemical & Pharmaceutical Co., Ltd., Taipei, Taiwan) and 10 IU human chorionic gonadotropin (hCG; Gona-500 X08; China Chemical & Pharmaceutical Co., Ltd., Taipei, Taiwan) at 48 h intervals. After injection of hCG, all mice were copulated by male mice. Mice with vaginal plugs on the following morning were regarded as embryonic day 0.5 (ED 0.5). The pronuclear embryos (ED 0.5) surrounded by cumulus cells were collected from the oviduct approximately 20 h after hCG injection and treated with 0.1% (w/v) hyaluronidase (Sigma-Aldrich, St. Louis, MO, USA) in drops of Hepes-Chatot-Ziomek-Bavister-Glucose (HCZBG) medium until cumulus cells dispersed. The pronuclear embryos were then transferred into new drops of HCZBG medium, and cumulus cells were removed completely by gentle pipetting. Embryos with intact pronuclei were selected and assigned randomly into drops of KSOM (KSOM-AA, MR-121D, Millipore, Billerica, MA, USA) medium containing 0, 25, 50, or 100 µg/mL of NSP. The drops of KSOM containing embryos were covered with mineral oil and cultured at 37°C in an atmosphere of 5% CO_2_ in air. At each stage (pronuclear, 2-cell, 4-8-cell, morula, and blastocyst), the morphology of embryos in each group was observed by reverted microscope. The embryos with even and regular cells and without fragmentation were regarded as high quality.

### Collection of embryos derived from mice consumed with NSP by intragastric intubation

In toxicity study, female CD1 mice weighed 25 g were randomly divided into four groups. Each group of mice was given 0.2 mL of deionized and distilled water (DDW) containing 0, 25, 50, or 100 µg NSP once a day by the intragastric intubation method, respectively. During 1 week of NSP consumption, all mice were administered the superovulation procedure during the final two days, and ED 0.5 embryos were collected as described above but cultured in drops of KSOM medium without NSP. The method to exam embryo quality was depicted as mentioned previously.

### Assessment of blastocyst quality by immunostaining

After the embryos reached the blastocyst stage (96 h of culture), 4,6-diamidino-2-phenylindole (DAPI; 0.2 µg/mL; Molecular Probes, Inc., Eugene, OR, USA) staining and immunostaining for CDX2 (BioGenex, Inc., San Ramon, CA, USA) were applied to estimate the total cell numbers and the cell numbers of trophectoderm (TE) in the blastocysts, respectively. After removing the zona pellucida with phosphate buffered saline (PBS), pH 2 containing 1% (w/v) bovine serum albumin (BSA), the blastocysts were then fixed in 4% (w/v) paraformaldehyde in PBS for 40 min at room temperature, washed twice with blocking solution (1% (w/v) BSA in PBS containing 0.1% (v/v) Tween-20 (Sigma-Aldrich, St. Louis, MO, USA)), and permeabilized with PBS containing 0.3% (v/v) Triton X-100 (Nacalai Tesque, Inc., Kyoto, Japan) for 20 min at room temperature. After blocking with blocking solution for 1 h at room temperature, the blastocysts were incubated with anti-CDX2 mouse monoclonal antibody, diluted in blocking solution (1∶200), overnight at 4°C. The next morning, the blastocysts were washed twice in blocking solution and then incubated with goat anti-mouse IgG-PE (Santa Cruz Biotechnology, Inc., Dallas, Texas, USA) diluted in blocking solution (1∶200) for 1 h at room temperature. Blastocysts were then washed twice with blocking solution and stained with DAPI. The cell number of the inner cell mass (ICM) was estimated by subtracting the cell number of TE from the total cell number of the blastocyst.

### Assessment of blastocyst quality by TUNEL assay

The mouse blastocysts were collected, fixed and permeabilized as described previously, and then subjected to the terminal deoxynucleotidyl transferase dUTP nick end labeling (TUNEL) assay using the fluorescent *In Situ* Cell Death Detection Kit (Roche, Boehringer-Mannheim, Germany) according to the manufacturer's instructions. After the TUNEL reaction, blastocysts were washed three times with PBS containing 1% (w/v) BSA and stained with DAPI.

### *In vitro* adsorption assay

FB_1_ (Sigma-Aldrich, St. Louis, MO, USA) powder was dissolved in sterilized DDW and diluted to 200 ng/mL at a ratio 100∶1, 10∶1, and 1∶1 to NSP. The mixture was incubated at room temperature for 2 h with agitation. After incubation, the mixtures were centrifuged at 5,600×g for 10 min [22], and the supernatants were collected for FB_1_ concentration determination through MaxSignal Fumonisin ELISA Test Kit (Bioo Scientific, Austin, TX, USA).

### *In vivo* adsorption assay

At ED 7.5, the pregnant CD1 mice weighing 35–40 g were allocated randomly into four groups and consumed 0.2 mL of various solutions on both ED 7.5 and 8.5. The Ctrl group was intubated-fed with DDW, the NSP group was fed with 100 µg of NSP (2.5 mg/kg), the F20 group was fed with 500 µg of FB_1_ (12.5 mg/kg), and the FN20 group was fed with 500 µg of FB_1_+100 µg of NSP. One hour after the last intragastric intubation on ED 8.5, blood was drawn through the tail vein of the female mouse, mixed well with heparin (10 mg/mL, Sigma-Aldrich, St. Louis, MO, USA) for anticoagulation, and centrifuged at 3,000 rpm for 5 min. The plasma was collected and assayed for the concentrations of FB_1_ with the MaxSignal Fumonisin ELISA Test Kit (Bioo Scientific, Austin, TX, USA). All mice were sacrificed on either ED 10.5 or ED 17.5 to observe neural tube defects by stereomicroscopy. In addition, the fetuses and placentas were collected and weighed on ED 17.5.

### Investigation of gene expression of sphingolipid metabolism enzymes by quantitative real-time PCR

The pregnant mice from the four treatment groups, Ctrl, NSP, F20, and FN20, were sacrificed on ED 10.5, and their livers, uterus, fetuses, and placentae were removed. Total RNA was extracted from liver, uterus, fetus, and placenta samples and reverse-transcribed into cDNA. Quantitative real-time PCR was performed with the Applied Biosystems StepOne Real-time PCR system with SYBR green. The expression levels of *longevity assurance homolog 5* (*LASS5*) [23], *sphingosine kinase 1* (*Sphk1*), *sphingosine kinase 2* (*Sphk2*), *sphingosine 1- phosphate lyase* (*Sgpl1*), *sphingosine 1-phosphate phosphatase* (*Sgpp1*), and *Glyceraldehyde 3-phosphate dehydrogenase* (*GAPDH*) were detected with the primers listed in Table 1.

**Table 1:** The primer lists for quantitative real time PCR.

Gene	Accession	Primer Sequence	Length (bp)
*LASS5*	NM_028015.2	Forward: 5′-GCAATGGTGCCAACTGCAT-3′ Reverse: 5′-TCCCCTGCTCTTCAGCCA-3′	63
*Sphk1*	NM_011451.3	Forward: 5′-TCTGGGCTGCGGCTCTATT-3′ Reverse: 5′-AGGTCCACGTCAGCAACAAAG-3′	62
*Sphk2*	NM_203280.3	Forward: 5′-CGGCCCACGGTTTGC-3′ Reverse: 5′-GGGCGTAGTCGCTGTATGTGT-3′	60
*Sgpl1*	NM_009163.3	Forward: 5′-GTTGGGCCGCCTTGATG-3′ Reverse: 5′-GATGATCTGTTTGGTAGCTTCAACA-3′	62
*Sgpp1*	NM_030750.3	Forward: 5′-CCCATTGGTGGACCTGATTG-3′ Reverse: 5′-GATGAGCGGCGCATATTTG-3′	58
*GAPDH*	NM_008084.2	Forward: 5′-AACCTGCCAAGTATGATGACATCA-3′ Reverse: 5′-GGAAGAGTGGGAGTTGCTGTTG-3′	128

### Statistical analysis

Analysis of variance was performed with the General Linear Model procedure of SAS (SAS Enterprise Guide 4.1, SAS Institute Inc., Cary, North Carolina, USA). Significance of differences was analyzed by Duncan's multiple range test, χ^2^ test, and Fisher's exact test. P<0.05 was considered to be statistically significant.

## Results

### NSP did not disturb the development of intact pre-implantation mouse embryos

**Figure 1:**
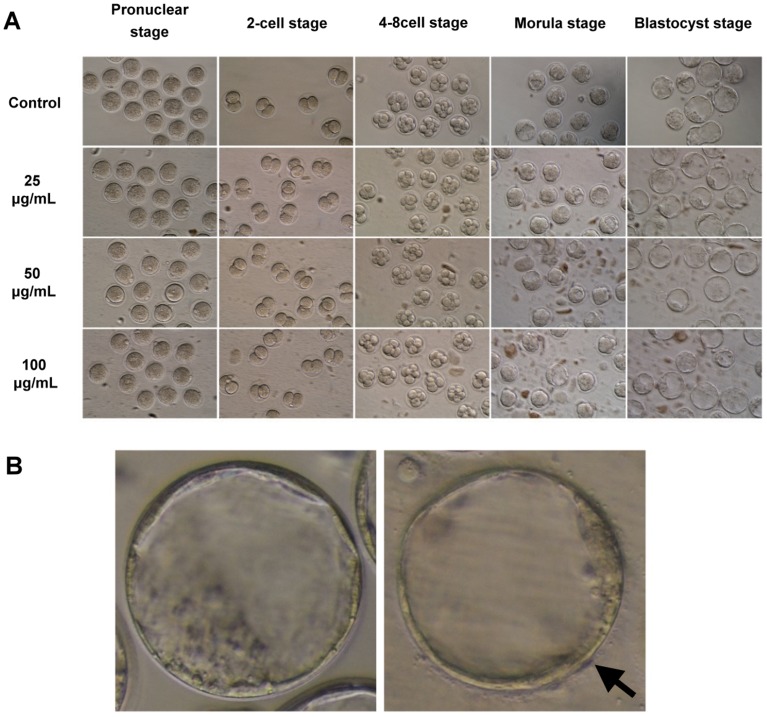
The effect of NSP on the development of intact pre-implantation mouse embryos *in vitro*. (A) Intact pre-implantation mouse embryos were collected from 32 mice and cultured in the medium containing various concentrations of NSP before morphological analysis. (B) NSP aggregation on the surface of the zonae pellucidae of mouse blastocysts. Arrow indicates the clusters of NSP (Left panel, control embryo; Right panel, embryo in 100 µg/mL of NSP).

**Figure 2:**
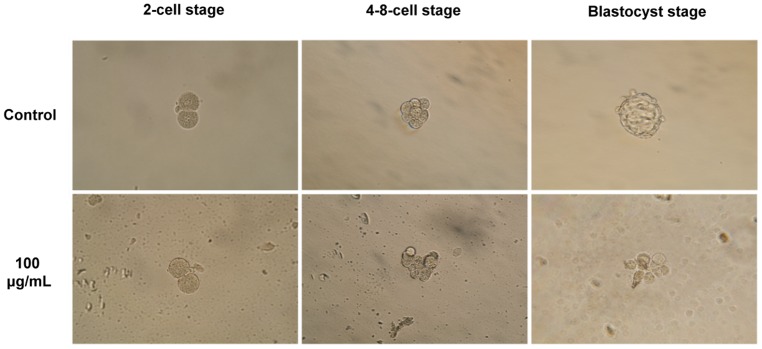
NSP disturbs the development of zona-free pre-implantation mouse embryos. Embryos were cultured in medium containing 100 µg/mL of NSP *in vitro* (embryos were collected from 12 mice).

To assess the toxicity of NSP on the development of pre-implantation mouse embryos, we treated embryos with different concentrations of NSP in the culture medium and then examined the morphology and development of embryos *in vitro* (embryos collected from 32 mice). The embryos with even and regular cells and without fragmentation were regarded as high quality. During the culture period from the pronuclear stage to the blastocyst stage, NSP tended to be deposited on the bottom of the culture dish (Fig. 1A) and associated with the surface of the zonae pellucidae of embryos (Fig. 1B), in a time- and dose-dependent manner. Treatment with NSP did not induce aberrant embryonic morphology (Fig. 1). Table 2 shows that the developmental rates of embryos from the pronuclear to the blastocyst stage were unaffected by the presence of NSP. We postulated that the zona pellucida might block any possible harmful effect of NSP on the embryonic development. The zona-free embryos were subjected to the same treatments as the intact embryos. Instead of aggregating on the zona pellucida, NSP accumulated on the embryonic surface, where it disturbed cell compaction during the formation of morulae or blastocysts (embryos collected from 12 mice) (Fig. 2).

**Table 2:** The effect of NSP on the development of intact pre-implantation mouse embryos cultured *in vitro*.

NSP (µg/mL)	No. pronuclear stage (%)	No. 2-cell stage (%)	No. 4-8-cell stage (%)	No. morula stage (%)	No. blastocyst stage (%)
Control	114 (100.0±0.0)	114 (100.0±0.0)	113 (99.1±1.9)	109 (95.6±2.2)	104 (91.2±2.3)
25	117 (100.0±0.0)	117 (100.0±0.0)	112 (95.7±1.9)	112 (95.7±1.9)	107 (91.5±3.8)
50	114 (100.0±0.0)	114 (100.0±0.0)	112 (98.3±1.2)	112 (98.3±1.2)	104 (91.2±3.5)
100	115 (100.0±0.0)	115 (100.0±0.0)	113 (98.3±0.9)	111 (96.5±1.4)	107 (93.0±3.2)

No significant differences were observed.

NSP was deposited in chick liver and kidney, and some might be excreted via feces after feed trial [24]. Therefore, we speculate that NSP might possibly affect reproductive system through circulation system. Because the *in vitro* assay showed no significant changes in the pre-implantation development of intact mouse embryos, we next investigated the morphology of embryos after the mother mice were intragastric intubated NSP. Four groups of mice were given 0.2 mL of DDW containing different doses of NSP (0, 25, 50, and 100 µg) daily via the intragastric tube for 1 week (64 mice total). On day 5 of the treatment period, the superovulation procedure was conducted, and embryos were collected on ED 0.5. These pronuclear embryos were cultured in KSOM medium without NSP from the pronuclear stage to the blastocyst stage to investigate the embryonic development *in vitro*. Similar morphology and developmental rates were observed among the groups (Fig. S1 and Table S1).

### NSP has no impact on the quality of mouse blastocysts

**Figure 3:**
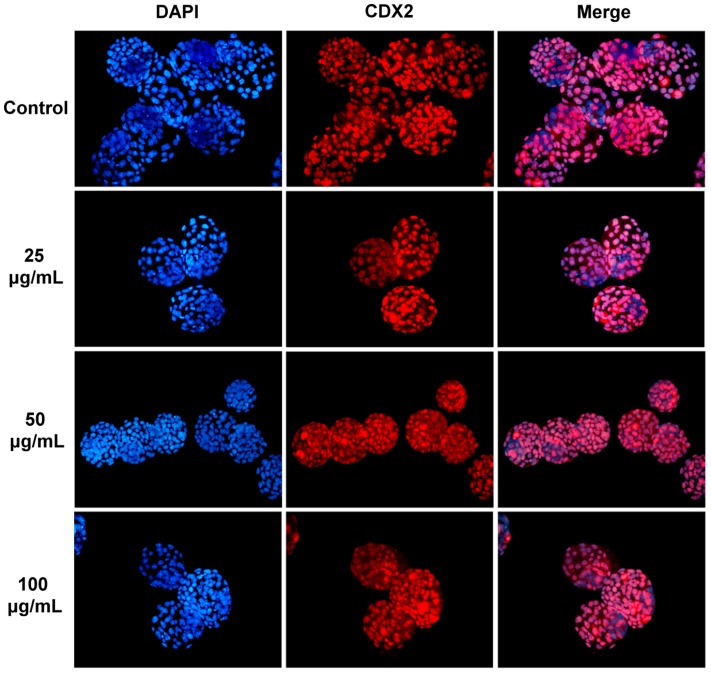
The total cell number in mouse blastocysts. Blastocysts were derived from pronuclear embryos cultured in different doses of NSP. The nuclei in the blastocysts were stained with DAPI. Trophectoderm cells were counted via immunocytochemical staining for CDX2.

**Figure 4:**
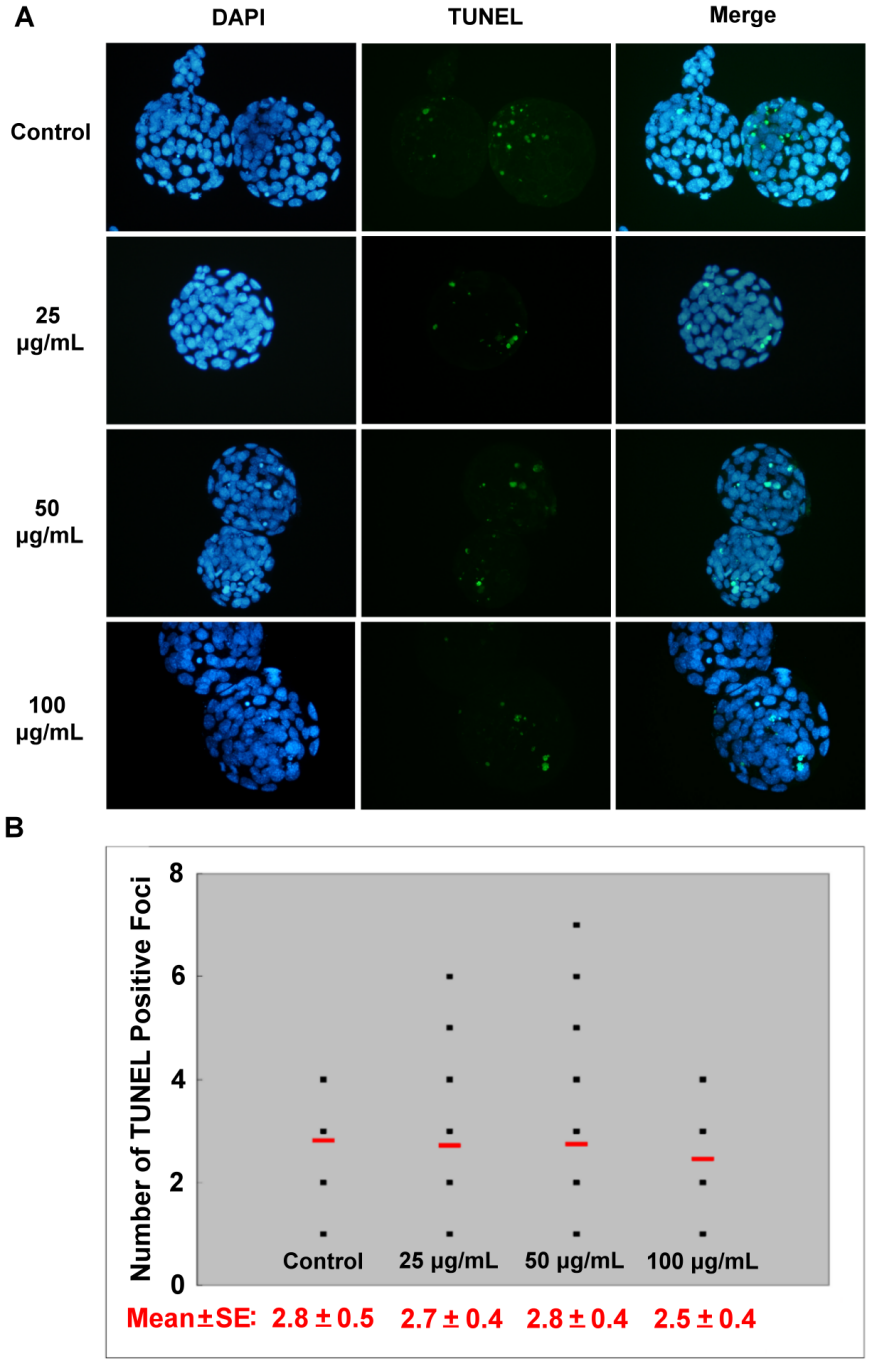
The apoptotic cells in mouse blastocysts. Blastocysts were derived from pronuclear embryos cultured in different doses of NSP. (A) The nuclei in the blastocyst were stained by DAPI, and the incidence of apoptosis was detected by TUNEL assay. (B) The average number of TUNEL-positive cells in each treatment group is indicated by the short horizontal bar.

Although no significant defects in the morphology and development were observed in the mouse embryos cultured in the NSP-containing medium from the pronuclear to the blastocyst stage, the quality of embryos was further evaluated. The quality of blastocysts was examined by counting the total cell numbers in blastocysts (by DAPI staining), the expression pattern of TE marker (CDX2 staining), and the cell number in the ICM (subtracting the TE cell number from the total cell number), as shown in Figs. 3 and S2. The total numbers of blastocyst nuclei, TE cells, and ICM were not significantly different among the control and NSP-treated groups (Table 3). There were also no significant differences when these embryos were compared with those derived from females intubated-fed with NSP (Table S2). This suggested that NSP has no negative effects on cell nuclei or ICM cell numbers during the pre-implantation development of mouse embryos *in vitro*. We next examined the incidence of apoptosis in the blastocysts. These results also showed no significant difference in any treatment (Fig. 4). Furthermore, similar results were demonstrated in the embryos derived from the female mice intubated-fed with NSP (Fig. S3). Based on these results, we concluded that NSP did not cause any significant deleterious effect on the development of pre-implantation mouse embryos *in vitro*. We further assessed the adsorption of NSP on FB_1_ by *in vitro* and *in vivo* assays.

**Table 3:** The cell number of mouse blastocysts derived from the pronuclear embryos cultured in medium containing NSP *in vitro* a.

NSP (µg/mL) (n)&	Total cell number by DAPI staining	Number of TE* cells by CDX2 staining	Number of ICM# cells^b^
Control (18)	62.4±3.7	48.2±4.1	14.2±1.2
25 (22)	63.4±1.9	48.7±2.3	14.6±1.4
50 (21)	63.8±2.2	49.4±2.4	14.4±1.2
100 (19)	63.3±2.5	49.8±3.0	14.1±0.9

*TE, trophectoderm;

# ICM, inner cell mass;

& n, number of blastocysts.

a No significant differences were observed.

b Number of ICM cells was estimated by subtracting the TE cell numbers from the total cell numbers.

### Fumonisin B_1_ was adsorbed by NSP *in vitro* and *in vivo*

**Figure 5:**
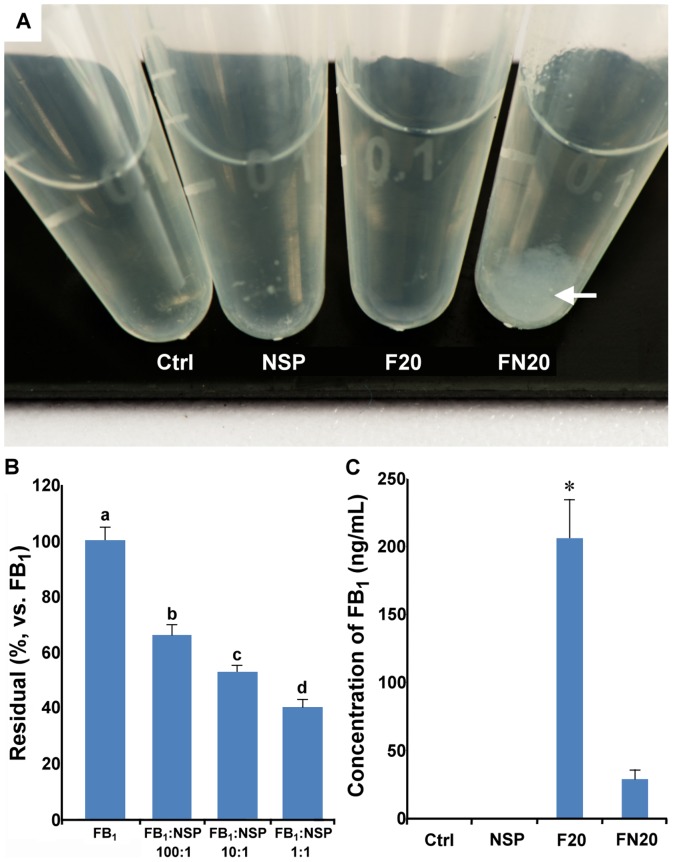
The *in vitro* and *in vivo* assays for FB_1_ adsorption by NSP. (A) A muddy phenomenon (arrow) was noticed after mixing FB_1_ and NSP. Ctrl, tube containing DDW; NSP, tube containing 100 µg/mL of NSP; F20, tube containing 500 µg of FB_1_; FN20, tube containing 500 µg of FB_1_+100 µg of NSP. (B) After 2 h incubation, the supernatant collected from the mixture in each treatment was assayed for the concentration of FB_1_ using MaxSignal Fumonisin ELISA Test Kit, and the adsorption of FB_1_ with NSP showed a significant non-linear relationship. (C) The 16 pregnant female mice were divided into four groups and were consumed 0.2 mL of DDW (Ctrl), 100 µg of NSP (NSP), 500 µg of FB_1_ (F20), or 500 µg of FB_1_+100 µg of NSP (FN20) on days 7.5 and 8.5 of pregnancy. The plasma was collected from blood drawn through the tail vein 1 h after the last consumption, and the concentrations of FB_1_ were analyzed using the MaxSignal Fumonisin ELISA Test Kit. ^*^: P<0.05 (Duncan's multiple range test).

NSP, prepared by the exfoliation of natural MMT clay, was used to absorb mycotoxins. Based on the reports of their strong binding, in which mixing FB_1_ and NSP results in a cloudy solution (Fig. 5A), we expected that NSP would be useful for mycotoxin adsorption. To address this hypothesis, mixed solutions containing FB_1_ and NSP were analyzed in both an *in vitro* assay and an intubation trial of FB_1_ and NSP in female CD1 mice. In the *in vitro* assay, the adsorption of FB_1_ with NSP showed a significant non-linear relationship (Fig. 5B, P<0.05), in which FB_1_:NSP at 1∶1 ratio can reached about 50% absorption rate. In the *in vivo* assay, pregnant mice were given 0.2 mL solution containing Ctrl, NSP, F20, and FN20, respectively, on ED 7.5 and 8.5. One hour after the last intubation on ED 8.5, blood was drawn through the tail vein, and the residual FB_1_ in blood was analyzed. These results indicated a much higher reduction rate than that shown in the *in vitro* assay (total 16 mice were used) (Fig. 5C, P<0.05).

### Negative effects caused by FB_1_ were ameliorated by NSP

**Figure 6:**
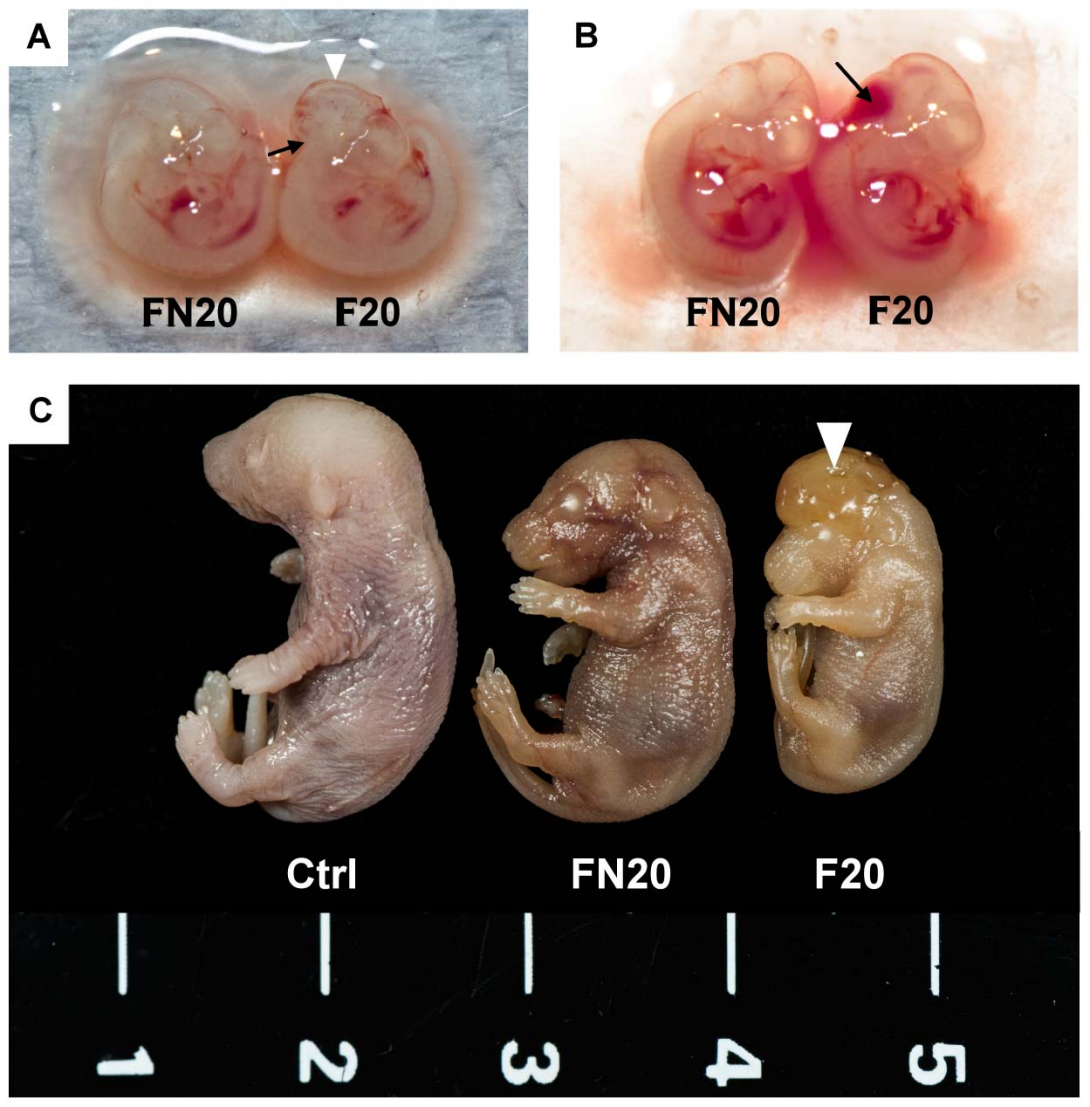
Fetal morphology from FB_1_-treated mothers. Female mice were intubated-fed with 500 µg of FB_1_ (F20) or 500 µg of FB_1_+100 µg of NSP (FN20) on days 7.5 and 8.5 of pregnancy. (A) At ED 10.5, a mouse fetus with a slight neural tube defect on hindbrain (arrow) and a hemorrhage on the surface of brain (triangle) were found in the F20 group (n = 5 pregnant mice). (B) Acute hemorrhage (arrow) was found on the hindbrain of an ED 10.5 fetus from the F20 group. (C) Exencephaly (triangle) was observed in an ED 17.5 fetus derived from a female mouse intubated-fed with F20 (n = 7 pregnant mice), and this fetus was smaller than those from the control and FN20 group (n = 5 pregnant mice).

**Figure 7:**
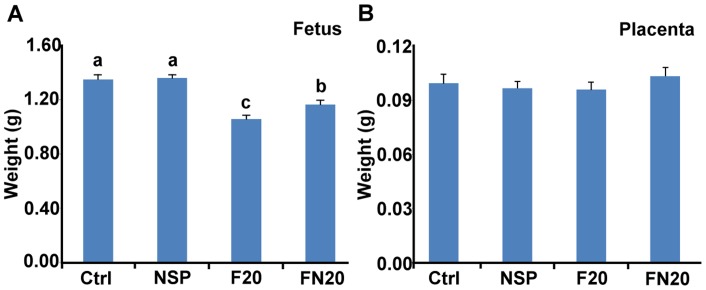
Weights of fetuses and placentae on ED 17.5. Weights of fetuses (A) and placentae (B) from the ED 17.5 conceptuses derived from the 12 mice intubated-fed with DDW (Ctrl) (20 offspring), 100 µg of NSP (NSP) (36 offspring), 500 µg of FB_1_ (F20) (28 offspring), or 500 µg of FB_1_+100 µg of NSP (FN20) (22 offspring), respectively, on days 7.5 and 8.5 of pregnancy. ^abc^: P<0.05 (Duncan's multiple range test).

**Figure 8:**
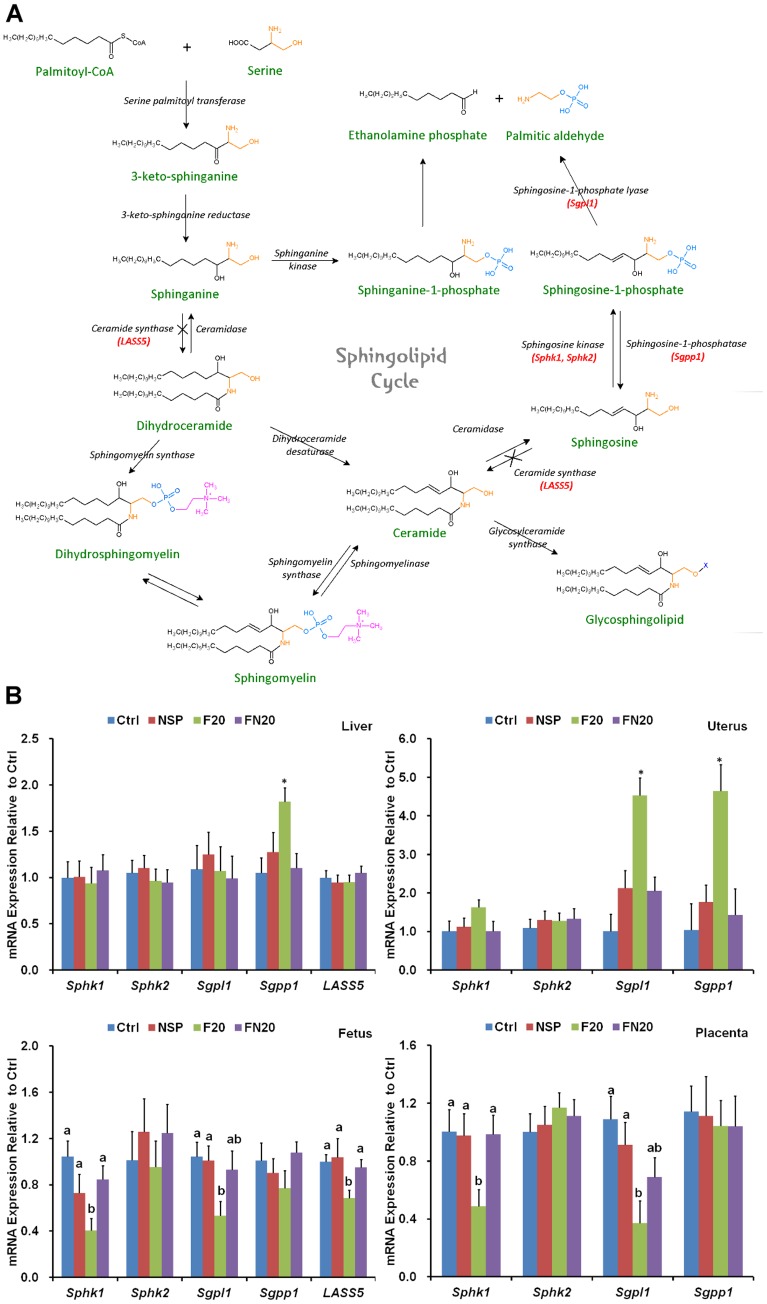
Inhibition of the ceramide synthesis pathway by FB_1_. (A) The ceramide synthesis pathway could be blocked by FB_1_ (indicated by ×). (B) The 20 pregnant mice were divided into four groups and were intubated-fed with DDW (Ctrl), 100 µg of NSP (NSP), 500 µg of FB_1_ (F20), or 500 µg of FB_1_+100 µg of NSP (FN20) on days 7.5 and 8.5 of pregnancy. They were sacrificed on ED 10.5, and the gene expression of *LASS5*, *Sphk1*, *Sphk2*, *Sgpl1*, and *Sgpp1* was investigated by quantitative real-time PCR. ^*^: P<0.05 (Duncan's multiple range test).

In addition to the *in vitro* and *in vivo* adsorption assays for evaluating the residual amount of mycotoxin, the adsorption efficiency of NSP was determined by animal experiments. To evaluate whether the negative effects caused by FB_1_ were reduced by NSP, pregnant mice were intubated-fed with 0.2 mL solution containing Ctrl, NSP, F20, and FN20, respectively, on ED 7.5 and 8.5. On ED 10.5, mice were sacrificed, and the fetuses were collected for morphological examination. The fetuses showed minor neural tube defects at the hindbrain level, and brain hemorrhages were discovered on the brain surface (n = 1, in total 40 fetuses; Fig. 6A), especially on the hindbrain (n = 2, in total 40 fetuses; Fig. 6B) in the F20 group (4 pregnant mice examined). However, neural tube defects and brain hemorrhages were not observed in the FN20 group (n = 0, in total 35 fetuses, 3 pregnant mice examined). Because the fetal development is almost completed at ED 17.5, we also dissected the fetuses on ED 17.5. Obvious exencephaly was found in fetuses derived from the female mice intubated-fed with F20 (Fig. 6C) (7 pregnant mice examined), although the incidence of exencephaly was only 7.4% (Table 4). Moreover, the fetal weight was significantly decreased in the F20 group (n = 28 fetuses, P<0.05) and was restored by supplementation with NSP (n = 22 fetuses) (Fig. 7A). The placental weight showed no significant differences in all treatments (Fig. 7B). FB_1_ was a strong inhibitor of ceramide synthase (Fig. 8A), and may also disturb the metabolism of the sphingolipids that are important for stabilizing the structure and function of the cell membrane (Fig. 8A) [23], [25], [26]. To evaluate whether the inhibition by FB_1_ could be reduced by NSP, samples of the maternal liver, uterus, fetus, and placenta were collected on ED 10.5 after tube-feeding, and the gene expression of *LASS5*, *Sphk1*, *Sphk2*, *Sgpl1*, and *Sgpp1* was investigated by quantitative real-time PCR (Fig. 8B). In the liver, the expression of *LSAA5*, *Sphk1*, *Sphk2*, and *Sgpl1* was normal, but the expression of *Sgpp1* was significantly increased after treatment with F20 (n = 5 treated mice). However, the expression was restored by supplementation with NSP (n = 5 treated mice). In the uterus, the expression of *Sphk1* and *Sphk2* was normal, but the expression of *Sgpl1* and *Sgpp1* was significantly increased after treatment with F20 (n = 5 treated mice). However, the expression was also restored by supplementation with NSP (n = 5 treated mice). In the fetus and placenta, the expression of *Sphk2* and *Sgpp1* was normal, but the expression of *Sphk1* and *Sgpl1* was significantly attenuated. The reduced expression of *LASS5* was also shown in fetus after treatment with F20 (n = 5 treated mice). Again, the expression was restored by supplementation with NSP (n = 5 treated mice) (Fig. 8B).

**Table 4:** The incidence of exencephaly in ED 17.5 mouse fetuses*.

	Ctrl	NSP	F20	FN20
No. dams	6	5	7	5
No. dams (Ex) 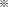	0	0	4	1
No. fetuses	80	85	108	57
No. early death	1	3	4	1
No. exencephaly	0^a^	0^a^	8^bc^	1^ac^
% exencephaly#	0^a^	0^a^	7.4^bc^	1.8^ac^

*The fetuses were derived from females consumed DDW (control; Ctrl), 100 µg of NSP (NSP), 500 µg of FB_1_ (F20) or 500 µg of FB_1_+100 µg of NSP (FN20) on days 7.5 and 8.5 of pregnancy.

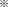
Number of dam having exencephalic fetus.

# % exencephaly  =  No. exencephaly/No. fetuses ×100.

abc P<0.05 (Fisher's exact test).

## Discussion

Corn and soybean meal are major ingredients in animal feed. Corn is also the best medium to support *Fusarium verticillioides* growth. Almost all of the hog, broiler, and layer feed in Taiwan is reported to be contaminated by FB_1_ at up to 1.3 mg/kg on average [11]. Therefore, how to prevent and reduce the deteriorated effects caused by FB_1_ is an important problem. Ceramide synthase can use sphinganine or sphingosine with fatty acyl-CoA to synthesize ceramide (Fig. 8A) [8]. Ceramide is a critical intermediate product during sphingolipid metabolism, producing sphingomyelin, sphingosine, or glycosphingolipid. However, due its chemical similarities with sphinganine and sphingosine, FB_1_ may inhibit the activity of ceramide synthase localized at the endoplasmic reticulum and disturb the metabolism of the sphingolipids that are important for stabilizing the structure and function of the cell membrane (Fig. 8A) [23], [25], [26]. In addition, free sphingoid bases were accumulated because of inhibition of ceramide synthase. The inhibition of ceramide synthase can promote free sphingoid base-induced cell death but inhibit cell death triggered by ceramide. Cells that are sensitive to sphingoid base-induced cell death will die and insensitive cells will survive [26]. It has been hypothesized that FB_1_ can decrease the production of glycosphingolipids and thus lead to impaired function of the folate transporters on the cell membrane, and the intake of folate decreases after exposure to FB_1_ [27], [28], [29], [30]. The low folate intake is responsible for the increased incidence of neural tube defects and the failure of neurulation during embryogenesis, particularly the exencephaly [9]. However, a recent study held the opposite opinion which demonstrated that folate deficiency does not exacerbate NTD induction by FB_1_ in LM/Bc mice [31].

Although the level of mycotoxin contamination in feed could be reduced by proper management during feed production, it is very difficult to eradicate mycotoxins. Therefore, feed additives, such as mycotoxin binders, are used in animal feed to eliminate the activity of mycotoxins. Mycotoxin binders can be divided into mycotoxin-adsorbing agents and mycotoxin-transforming agents. The former can adsorb mycotoxins in the gastrointestinal tracts and form complexes to excrete, whereas the latter can reduce the toxicity of mycotoxins by degrading mycotoxins into non-toxic structures [12]. Aluminosilicates are the largest group of mycotoxin adsorbing agents, and they include bentonites, MMT, zeolites, and HSCAS. The NSP (ca. 80×80×1 nm^3^) used in this study was exfoliated from natural MMT and possessed huge surface area (ca. 720 m^2^/g) and high ion density (ca. 20,000 ions/platelet) [16], [17]. These unique characteristics showed excellent microorganism-binding activity [18], [19], [20]. These previous results inspired us to investigate whether NSP could adsorb FB_1_ *in vitro* and *in vivo* to ameliorate the negative effects on embryonic development. Before evaluating the ability of NSP to adsorb FB_1_, we assessed the toxicity of NSP on the development of mouse embryos. These results indicated that NSP would not inhibit the development of intact pre-implantation mouse embryos, although NSP aggregated on the surface of the zona pellucida during *in vitro* culture (Fig. 1B). Early embryos hatching from zona pellucida may occur occasionally. Hence, the zona-free embryos were also examined in this study, and we found that NSP could hinder the development of zona-free embryos (Fig. 2). Furthermore, the embryos derived from the female mice intubated-fed with NSP were able to develop normally to the blastocyst stage (Fig. S1). Ten NSP-treated females per dose were allowed to give birth, and the appearance and growth of the offspring did not differ from those offspring from 8 control litters. Although changes of weight were observed in the NSP-treated females during the consumption period, these were considered likely to be normal day-to-day fluctuations (data not shown). Based on the *in vitro* and *in vivo* study, embryos could still develop successfully even if NSP deposited in the oviduct or uterus, due to the protection from the zona pellucida.

Specific features of the adsorbants, *i.e.*, total charge and charge distribution, pore size, accessible surface area, and adsorption affinity to mycotoxins, are the critical factors determining the adsorption efficiency [12]. Aluminosilicates are rich in negative charges, allowing them to adsorb mycotoxins in the gastrointestinal tracts of animals [12], [13], and they are highly effective at adsorbing aflatoxins but are limited for other mycotoxins [14]. The NSP was exfoliated from MMT, and FB_1_ was selected to evaluate whether NSP possesses adsorbing ability. After mixing NSP and FB_1_, a muddy phenomenon was observed, and it was suggested that FB_1_ was adsorbed by NSP (Fig. 5A,B). The residual concentration of FB_1_ in blood was also significantly decreased by supplementation with NSP in the consumption assay (Fig. 5C). In the *in vivo* assay, some organisms in the gastrointestinal tract of animals might degrade FB_1_, or there may be other specific pathways facilitating its *in vivo* adsorption by NSP.

A further investigation was conducted to study the *in vivo* adsorption efficiency of NSP on FB_1_. We examined the incidence of neural tube defects and weight changes of fetus and placenta, as well as the gene expression of *LASS5* (in maternal liver and fetus), *Sphk1*, *Sphk2*, *Sgpl1*, and *Sgpp1* in the maternal liver, uterus, fetus, and placenta. These results showed exencephaly in 7.4% of ED 17.5 fetuses derived from the female mice intubated-fed with 500 µg of FB_1_ on ED 7.5 and ED 8.5 (Table 4), compared with 1.8% in the group of mice intubated-fed 500 µg of FB_1_+100 µg of NSP. Among those seven FB_1_-treated female mice, four had fetuses with NTD (Table 4). In addition, ED 17.5 fetuses derived from female mice treated with F20 showed decreases in weight, but the placental weight was not changed (Fig. 7). It has been reported that the incidence of exencephaly in the strain of LM/Bc mice was approximately 80% under the same treatment dosage of FB_1_ used in the present study [7], [10].

Furthermore, another study showed that FB_1_ was gavaged daily to CD1 mice from ED 7 through ED 15 did not cause NTD, except at the dose of 25 mg/kg or more [32]. However, NTD was observable at the dose of 12.5 mg/kg in present study. This might be due to random chance and no significant difference between F20 and FN20 groups. Even though the strain of CD1 mice is less sensitive to FB_1_ than the LM/Bc strain is, NSP induced an observable reduction in the defects caused by FB_1_.

In mammalian cells, six isoforms of ceramide synthase exist: LASS1, LASS2, LASS3, LASS4, LASS5, and LASS6. The functions of LASS1, LASS4, and LASS5 are known, but the physiological roles of LASS2, LASS3, and LASS6 is unknown yet [23]. *LASS5* is expressed ubiquitously in murine tissues and is the major ceramide synthase gene in sphingolipid metabolism. In addition, a previous study showed that the ceramide synthase activity of murine lung epithelia with or without *LASS5* expression plasmid transfection was reduced by FB_1_ [23], and the (Dihydro) ceramide synthase activity of purified LASS5 was also inhibited by FB_1_ [33]. Therefore, we assumed that FB_1_ might interact with *LASS5* at the genetic level. In the present study, the gene expression of *LASS5* was significantly inhibited by FB_1_ in the fetus but was restored by supplementation with NSP (Fig. 8B). Based on this result, it is possible that *LASS5* might be a target for FB_1_, but further studies should be performed to clarify the importance of other isoforms of ceramide synthase. On the other hand, the gene expression of sphingolipid metabolism enzymes in maternal liver was not significantly different, except *Sgpp1*. However, increased expression of *Sgpl1* and *Sgpp1* was obvious in uterus (Fig. 8B). These results might disturb the metabolism of sphingosine-1-phosphate. Moreover, the expression patterns in fetus were similar to that in placenta, and these results might imply the presence of placental-fetal hormonal interactions. Further studies should be performed to reveal the expression of sphingolipid. The results of the present study indicated that FB_1_ could be adsorbed and its negative effects ameliorated by NSP. In conclusion, in use as a strong adsorbing agent for mycotoxins, NSP had no adverse effect on the development of intact pre-implantation mouse embryos *in vitro*. The newborn mice produced by females intubated-fed with NSP showed no abnormalities. In adsorption assays, the high *in vitro* adsorption efficiency in our study was unexpected. Furthermore, the high *in vivo* reduction of FB_1_ by NSP was confirmed by the low residual rate of FB_1_ in blood, the reduced incidence of neural tube defects, the increased fetal weight, and the restored gene expression of sphingolipid metabolism enzymes. Thus, we expect that the use of NSP as a supplement in animal feed may be an appropriate approach to minimize the toxicity of FB_1_ to animals.

## Supporting Information

Figure S1 **The development of intact pre-implantation mouse embryos cultured** ***in vitro*** **.** The pronuclear embryos derived from the female mice which had been fed with different doses of NSP for 1 week were cultured in KSOM medium without NSP to the blastocyst stage *in vitro*.
(TIF)

Figure S2 **The total cell number of mouse blastocysts.** The blastocysts were derived from the female mice which had been fed with different doses of NSP for 1 week. The pronuclear embryos were collected from the NSP-fed mice and cultured in KSOM medium without NSP to the blastocyst stage *in vitro*. The nuclei in the blastocyst were stained by DAPI. By immunocytochemical staining of CDX2, the trophectoderm cell was able to count.
(TIF)

Figure S3 **The apoptotic cells in mouse blastocysts.** The blastocysts were derived from the female mice which had been fed with different doses of NSP for 1 week. The pronuclear embryos were collected from the NSP-fed mice and cultured in KSOM medium without NSP to the blastocyst stage *in vitro*. (A) The nuclei in the blastocyst were stained by DAPI, and the incidence of apoptosis was detected by TUNEL assay. (B) The average of TUNEL positive cells in each treatment group are indicated by the short horizontal bar.
(TIF)

Table S1 The development of intact pre-implantation mouse embryos^*^ *in vitro*.
(DOC)

Table S2 The cell number of mouse blastocysts derived from the pronuclear embryos^*^ cultured *in vitro*.
(DOC)
