# Supplementary figures and images for: Inhibition of Fumonisin B1 Cytotoxicity by Nanosilicate Platelets during Mouse Embryo Development
Source: PLoS One. 2014 Nov 10;9(11):e112290. doi: 10.1371/journal.pone.0112290 (PMC4226500; doi:10.1371/journal.pone.0112290)

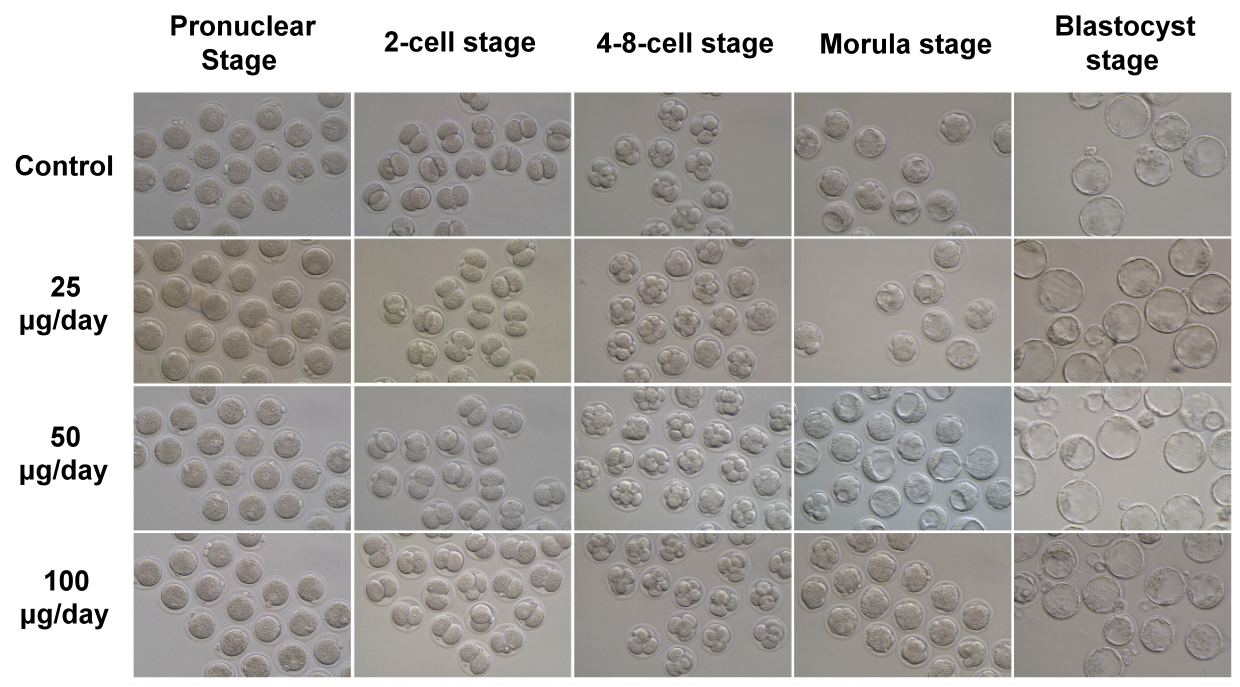

Supplement: Figure S1 — The development of intact pre-implantation mouse embryos cultured in vitro . The pronuclear embryos derived from the female mice which had been fed with different doses of NSP for 1 week were cultured in KSOM medium without NSP to the blastocyst stage in vitro. (TIF) [file pone.0112290.s001.tif]

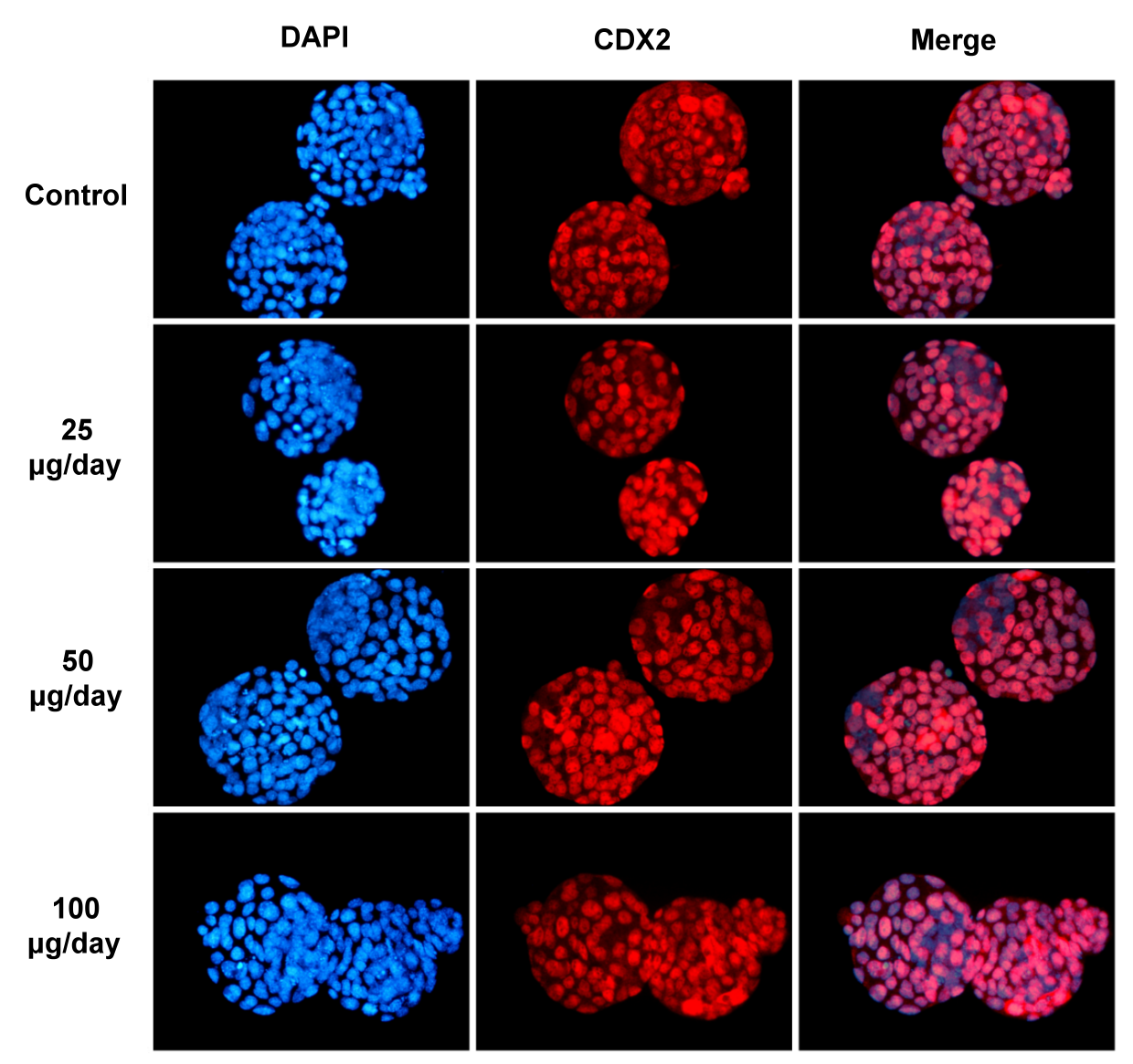

Supplement: Figure S2 — The total cell number of mouse blastocysts. The blastocysts were derived from the female mice which had been fed with different doses of NSP for 1 week. The pronuclear embryos were collected from the NSP-fed mice and cultured in KSOM medium without NSP to the blastocyst stage in vitro. The nuclei in the blastocyst were stained by DAPI. By immunocytochemical staining of CDX2, the trophectoderm cell was able to count. (TIF) [file pone.0112290.s002.tif]

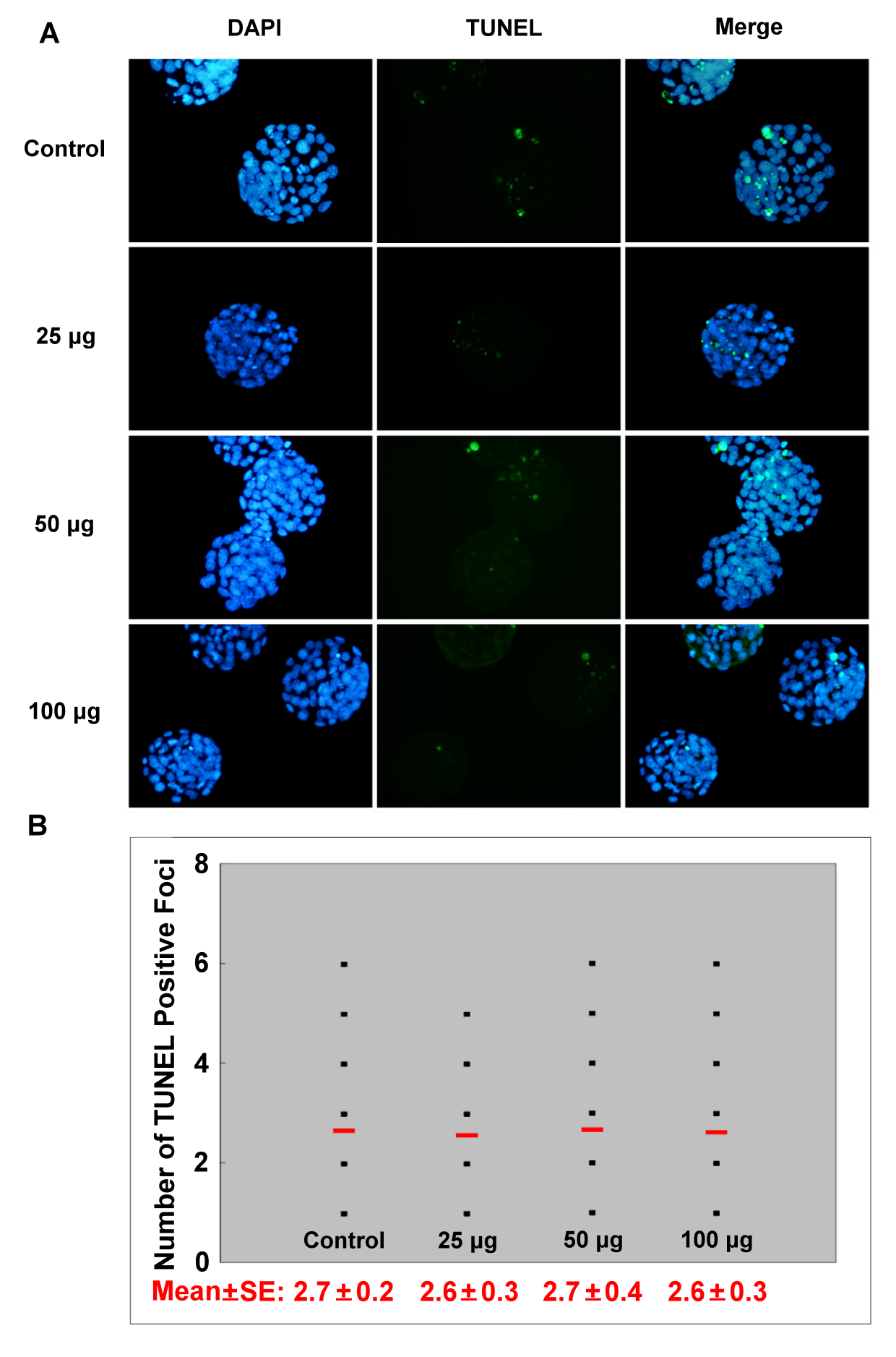

Supplement: Figure S3 — The apoptotic cells in mouse blastocysts. The blastocysts were derived from the female mice which had been fed with different doses of NSP for 1 week. The pronuclear embryos were collected from the NSP-fed mice and cultured in KSOM medium without NSP to the blastocyst stage in vitro. (A) The nuclei in the blastocyst were stained by DAPI, and the incidence of apoptosis was detected by TUNEL assay. (B) The average of TUNEL positive cells in each treatment group are indicated by the short horizontal bar. (TIF) [file pone.0112290.s003.tif]
