# Supplementary material for: Inhibition of Fumonisin B1 Cytotoxicity by Nanosilicate Platelets during Mouse Embryo Development
Source: PLoS One. 2014 Nov 10;9(11):e112290. doi: 10.1371/journal.pone.0112290 (PMC4226500; doi:10.1371/journal.pone.0112290)
Supplement: Table S1 — The development of intact pre-implantation mouse embryos* in vitro. (DOC) [file pone.0112290.s004.doc]

**Supporting Information**

**Table S1. The development of intact pre-implantation mouse embryos* *in vitro*.**

| **NSP^#^ (μg/day)** | **No. pronuclear stage (%)** | **No. 2-cell stage (%)** | **No. 4-8-cell stage (%)** | **No. morula stage (%)** | **No. blastocyst stage (%)** |
| --- | --- | --- | --- | --- | --- |
| **Control** | 169 (100.0 ± 0.0) | 169 (100.0 ± 0.0) | 164 (97.0 ± 2.5) | 155 (92.7 ± 4.0) | 153 (90.5 ± 3.8) |
| **25** | 139 (100.0 ± 0.0) | 139 (100.0 ± 0.0) | 138 (99.3 ± 0.6) | 132 (95.0 ± 0.7) | 130 (93.5 ± 1.0) |
| **50** | 179 (100.0 ± 0.0) | 179 (100.0 ± 0.0) | 178 (99.4 ± 0.6) | 169 (94.4 ± 2.3) | 165 (92.2 ± 4.2) |
| **100** | 141 (100.0 ± 0.0) | 141 (100.0 ± 0.0) | 140 (99.3 ± 0.4) | 131 (92.9 ± 4.9) | 128 (90.8 ± 5.3) |

* The pronuclear embryos derived from the female mice which had been fed with NSP by a feeding tube for 1 week were cultured in KSOM medium without NSP to the blastocyst stage *in vitro*.

^#^ The mice were fed with different doses of NSP for 1 week.

No significant difference in all treatments.
