# Supplementary material for: Inhibition of Fumonisin B1 Cytotoxicity by Nanosilicate Platelets during Mouse Embryo Development
Source: PLoS One. 2014 Nov 10;9(11):e112290. doi: 10.1371/journal.pone.0112290 (PMC4226500; doi:10.1371/journal.pone.0112290)
Supplement: Table S2 — The cell number of mouse blastocysts derived from the pronuclear embryos* cultured in vitro. (DOC) [file pone.0112290.s005.doc]

**Supporting Information**

**Table S2. The cell number of mouse blastocysts derived from the pronuclear embryos* cultured *in vitro*.**

| **NSP^#^ (μg/day) (n)^a^** | **Total cell number by DAPI staining** | **Number of TE^b^ cells by CDX2 staining** | **Number of ICM^c^ cells^d^** |
| --- | --- | --- | --- |
| **Control (40)** | 72.7 ± 1.7 | 58.6 ± 1.7 | 14.7 ± 0.5 |
| **25 (23)** | 72.3 ± 1.9 | 57.0 ± 1.8 | 14.7 ± 0.6 |
| **50 (32)** | 72.1 ± 2.2 | 58.8 ± 2.7 | 14.6 ± 0.6 |
| **100 (31)** | 72.3 ± 1.8 | 57.2 ± 1.8 | 14.7 ± 0.5 |

* The pronuclear embryos derived from the female mice which had been fed with NSP by a feeding tube for 1 week were cultured in KSOM medium without NSP to the blastocyst stage *in vitro*.

^#^ The mice were fed with different doses of NSP for 1week.

^a^ n, number of blastocysts; ^b^ TE, trophectoderm; ^c^ ICM, inner cell mass. ^d^ Number of ICM cells was estimated by subtracting the TE cell numbers from the total cell numbers.

No significant difference in all treatments.
